# Differential Expression and Regulation by Activin of the Neurotrophins BDNF and NT4 During Human and Mouse Ovarian Development

**DOI:** 10.1002/dvdy.22252

**Published:** 2010-02-19

**Authors:** Andrew J Childs, Rosemary AL Bayne, Alison A Murray, Sarah J Martins Da Silva, Craig S Collins, Norah Spears, Richard A Anderson

**Affiliations:** 1 MRC Human Reproductive Sciences Unit, Centre for Reproductive Biology, The Queen's Medical Research InstituteEdinburgh, United Kingdom; 2 Centre for Integrative Physiology, University of EdinburghHugh Robson Building, George Square, Edinburgh, United Kingdom; 3 Division of Reproductive and Developmental Sciences, University of Edinburgh, Centre for Reproductive Biology, The Queen's Medical Research InstituteEdinburgh, United Kingdom

**Keywords:** neurotrophin, BDNF, NT4, ovary, oocyte, primordial follicle, activin

## Abstract

The *t*ropomyosin-*r*elated *k*inase (Trk) B neurotrophin receptor is essential for ovarian germ cell survival and primordial follicle formation, but the contributions of its ligands, brain-derived neurotrophic factor (BDNF) and neurotrophin-4 (NT4), are unknown. We have investigated their expression and regulation in developing human and mouse ovaries. *BDNF* expression increased with increasing gestation, expression of human *NTF4* and of both *Ntf5* and *Bdnf* in the mouse was unchanged. *Bdnf* expression was dramatically lower than *Ntf5* in the mouse, but levels were comparable in the human. Human fetal ovarian somatic cells expressed BDNF. Activin A selectively regulated *BDNF* and *Ntf5* expression in human and mouse, respectively, identifying an oocyte/somatic signaling pathway which might mediate the pro-survival effects of activin. These data reveal that expression and regulation of the TrkB ligands are differentially controlled in the developing ovaries of humans and mice, and identify BDNF as a potential regulator of germ cell fate in the human fetal ovary. Developmental Dynamics 239:1211–1219, 2010. © 2010 Wiley-Liss, Inc.

## INTRODUCTION

The early development of the mammalian ovary is characterized by sequential waves of mitotic germ cell proliferation, meiotic entry and arrest, and subsequent association of oocytes with somatic cells to form primordial follicles. Extensive germ cell death occurs throughout these periods (Baker,^1963^; Pepling and Spradling,^2001^), and assembly of oocytes into primordial follicles is necessary for their continued survival (Hirshfield,^1991^; Fulton et al.,^2005^). The molecular mechanisms regulating these processes remain largely unknown, but the targeted disruption of several signaling pathways in rodent models has revealed extensive and bi-directional intercellular communication between the somatic and germ cell compartments to be crucial in controlling the onset and extent of follicle formation (reviewed in Skinner,^2005^; Tingen et al.,^2009^).

Among the signaling pathways implicated in regulating oogenesis and follicle formation in the developing ovary, the neurotrophin signaling system appears to be of particular importance (Ojeda et al.,^2000^; Dissen et al.,^2002^,^2009^). The neurotrophins are a family of peptide growth factors which signal through ligand-specific *t*ropomyosin-*r*elated *k*inase (Trk) receptors and the common p75^NTR^ receptor (Bibel and Barde,^2000^). Within the nervous system, neurotrophins have diverse roles in regulating neuronal survival, proliferation, differentiation and migration in a cell type- and context-specific manner (Davies,^2000^; Bernd,^2008^). The expression of components of the neurotrophin signaling pathway is not restricted to neural tissues, however, indicating the existence of functional roles for neurotrophins in other organ systems (Tessarollo,^1998^). Much of the neurotrophin signaling apparatus is expressed within the developing rodent and human ovary and testis, with several components displaying developmentally regulated expression changes around the time of primordial follicle formation (Dissen et al.,^1995^; Anderson et al.,^2002^; Robinson et al.,^2003^; Paredes et al.,^2004^; Dissen et al.,^2009^).

Signaling through the TrkB neuroptrophin receptor is essential for normal ovarian development, as targeted disruption of *Ntrk2* (encoding the TrkB receptor) results in a phenotype of impaired ovarian germ cell survival, reduced primordial follicle numbers (Spears et al.,^2003^; Kerr et al.,^2009^) and arrest of developing follicles at the primary to secondary follicle stage (Paredes et al.,^2004^). Expression of the TrkB receptor is restricted to germ cells in the feto-neonatal ovaries of humans and rodents at comparable developmental stages (Dissen et al.,^1995^; Anderson et al.,^2002^; Spears et al.,^2003^; Paredes et al.,^2004^), and is detectable in increasing amounts in germ cells in the fetal mouse ovary in the days before birth and the onset of follicle formation (Spears et al.,^2003^). TrkB binds two major neurotrophic ligands, namely brain-derived neurotrophic factor (BDNF, encoded by the *Bdnf* gene) and neurotrophin-4 (NT4, the product of the *Ntf5* locus in rodents, and the *NTF4* gene in humans). Whereas the expression of transcripts encoding both of these factors has been reported in the feto-neonatal ovaries of both humans and mice, the relative levels of each have not been established, nor have developmental changes been investigated. Furthermore, whereas NT4 expression has been localized to the somatic cells of the developing human fetal ovary (Anderson et al.,^2002^), the distribution of BDNF expression within the human fetal ovary remains unknown.

The biological significance of the expression of both BDNF and NT4 in the developing ovary is unclear, but may indicate neurotrophin-mediated regulation of more than one aspect of ovarian development at the time of follicle formation. Differential effects of BDNF and NT4 on neuronal survival and synapse formation have been reported, despite both ligands acting through the same (TrkB) receptor (Conover et al.,^1995^; Minichiello et al.,^1998^; Fan et al.,^2000^; Kalb,^2005^). Given the essential role of TrkB in primordial follicle formation in the mouse and evidence for a similar function in the human, we have investigated the expression and regulation of its ligands BDNF and NT4 in the period leading up to and during primordial follicle formation. We report here on a detailed analysis of developmental changes in expression of these growth/survival factors in the developing human and mouse ovary. Furthermore, in light of evidence that germ cell-expressed activin A indirectly promotes the proliferation and survival of germ cells in the human fetal ovary by influencing neighboring somatic cells (Martins da Silva et al.,^2004^; Coutts et al.,^2008^; Childs and Anderson,^2009^), we have also investigated whether BDNF and NT4 may be candidates for activin-regulated somatic cell-derived modulators of germ cell survival in the developing human and mouse ovary.

## RESULTS AND DISCUSSION

### TrkB Ligands Are Differentially Expressed in the Developing Ovaries of Humans and Mice

**Fig. 1:**
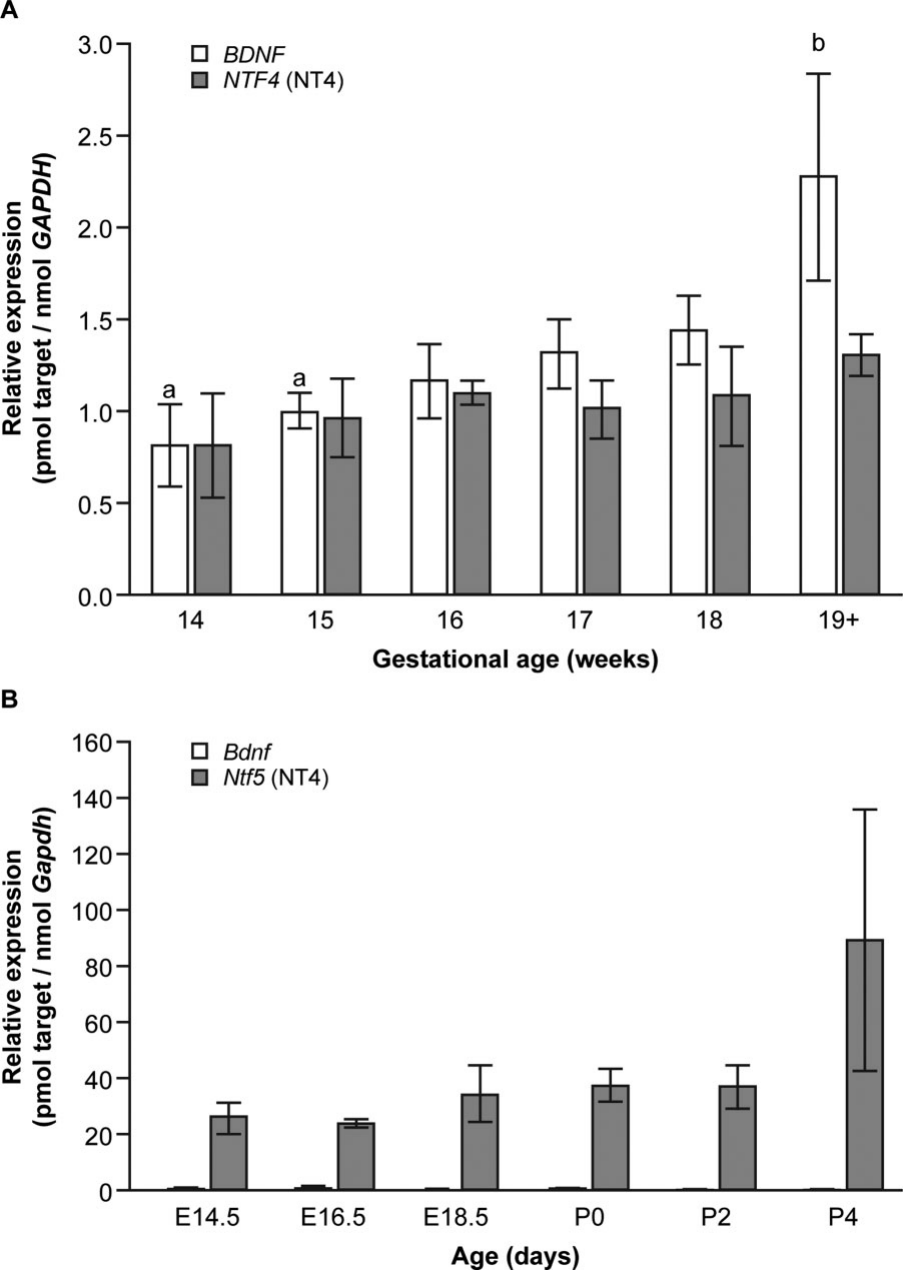
Expression of the genes encoding brain-derived neurotrophic factor (BDNF) and neurotrophin-4 (NT4) in the developing human and mouse ovary. **A**: Expression of *BDNF* and *NTF4* in the human fetal ovary. Expression of *BDNF* increased with gestation, and was significantly higher around the time of primordial follicle formation than at early gestations (a vs. b; *P* < 0.03). In contrast to the mouse, *BDNF* expression was comparable to that of *NTF4*. Expression of *NTF4* did not change significantly over gestation. **B**: Expression of *Bdnf* and *Ntf5* in the mouse feto-neonatal ovary from the onset of meiosis (embryonic day [E] 14.5) to primordial follicle formation (postnatal day [P] 2). *Ntf5* transcript levels rose slightly with increasing gestation, peaking around the onset of primordial follicle formation, but this was not significant. *Bdnf* levels were consistently very low at all gestations examined and showed no change over the period examined.

To establish the relative roles of TrkB ligands in oogenesis and follicle formation in the developing ovary, we first determined the expression profiles of transcripts encoding BDNF and NT4 in human and mouse ovaries at comparable developmental stages, from the initiation of meiosis to the onset of follicle formation. Both genes were expressed throughout this period in the human fetal ovary and standardization to the expression of the housekeeping gene glyceraldehyde-3-phosphate dehydrogenase (*GAPDH*) allowed comparison of relative expression between *BDNF* and *NTF4*. This revealed similar levels of expression of *BDNF* and *NTF4* at all gestations examined (Fig. 1A), although we detected differences in the expression of the two genes over the developmental window investigated. The expression of *BDNF* increased concomitant with the onset of primordial follicle formation in the human fetal ovary with levels of transcripts encoding BDNF significantly higher at 19 weeks gestation (2.27 ± 0.55 pmol *BDNF* transcript/nmol *GAPDH* transcript) than at 14 or 15 weeks (0.80 ± 0.22 pmol/nmol and 0.99 ± 0.10 pmol/nmol GAPDH, respectively, n = 5–6 per gestational group, *P* = 0.03), an approximately 2.5-fold increase over the gestational range examined. In contrast, expression of *NTF4* increased ∼1.6-fold over the gestational range examined, but this change was not statistically significant. It appears therefore that the expression of *BDNF*, but not that of *NTF4*, is developmentally regulated across mid-gestation in the human fetal ovary with an increase at the time primordial follicles are first seen.

In direct contrast to situation in the human, we found expression of *Ntf5* to be substantially higher than that of *Bdnf* in the mouse ovary across a comparable window in development (from embryonic day [E] 14.5 to postnatal day [P] 2), with the levels of transcripts encoding BDNF approximately 200 times lower than those encoding NT4 at P2. Expression of *Ntf5* appeared to increase gradually between E14.5 and P2, although this change did not reach statistical significance (26.0 ± 5.8 pmol/nmol rising to 89.6 ± 46.9 pmol/nmol *Gapdh*; *P* = 0.5; Fig. 1B), and no discernable change was observed in the expression of *Bdnf* over the gestational range examined. Notably, we failed to detect an increase in *Ntf5* expression around the time of follicle formation comparable to that reported previously in the neonatal rat ovary immediately after birth (Dissen et al.,^1995^). In the developing mouse ovary therefore, *Ntf5* appears to be the predominantly expressed TrkB ligand, whereas in the human both ligands are expressed at similar levels but only *BDNF* is developmentally regulated.

The finding that *Ntf5* expression is substantially higher than that of *Bdnf* in the mouse feto-neonatal ovary (up to ∼200× at P2) is consistent with the original description of high NT4 and low BDNF expression in *Xenopus* ovary, in contrast to all other tissues (Hallbook et al.,^1991^). This is striking given that expression from a single endogenous *Bdnf* allele is sufficient to maintain normal fertility in *Ntf5*^−/−^,*Bdnf*^+/−^ mice (Liu et al.,^1995^; although whether *Bdnf* expression from the only remaining allele is up-regulated to compensate has not been determined). Such differences in the expression of the two TrkB ligands may reflect a physiological requirement to balance the levels of the two ligands relative to each other within the developing ovary. Indeed, a possible context-dependent dosage effect of TrkB ligands has been reported in mice in which the coding sequence for NT4 has been knocked into the *Bdnf* locus (*Bdnf*^*nt4ki*^; Fan et al.,^2000^). Whereas mice carrying one knock-in allele and one null allele (*Bdnf*^*nt4ki*/−^) are normal and fertile, those that are homozygous for the knock-in allele (*Bdnf*^*nt4ki/nt4ki*^) are mostly infertile. This suggests that, although a small excess of NT4 in the absence of BDNF is tolerated, the production of NT4 from both *Bdnf* loci has a detrimental effect on fertility (Fan et al.,^2000^). Whether this phenotype arises in part from the two neurotrophins exerting differing biological potencies, as occurs in other organ systems (Minichiello et al.,^1998^; Kalb,^2005^), remains to be determined, however.

The absence of any change in the expression of both *NTF4* in the human, and *Ntf5* in the mouse across the period examined suggests that NT4 may not be a key regulator of follicle formation in either species; a hypothesis supported by the absence of a subfertility phenotype in homozygous NT4-deficient mice (Conover et al.,^1995^). This result differs from expression of transcripts encoding NT4 in the neonatal rat ovary, which have been reported to increase sharply but transiently in the hours after birth (Dissen et al.,^1995^). Only the expression of *BDNF* in the human fetal ovary appeared to be developmentally regulated across the gestational range examined, with expression being significantly higher at 19 weeks gestation than at 14 and 15 weeks. This increase in *BDNF* therefore correlates with the onset of primordial follicle formation in the human (∼18 weeks gestation). The small (∼two-fold) increase in *BDNF* expression with gestation reported here is likely to be below the sensitivity of recent whole-genome microarray studies investigating gene expression in the human fetal ovary around the time of primordial follicle formation (Fowler et al.,^2009^; Houmard et al.,^2009^), validating our quantitative reverse transcriptase-polymerase chain reaction (qRT-PCR) -based approach for detecting small changes in the expression of candidate genes relevant to this process.

### *BDNF* Is Predominantly Expressed by the Somatic Cells of the Human Fetal Ovary

**Fig. 2:**
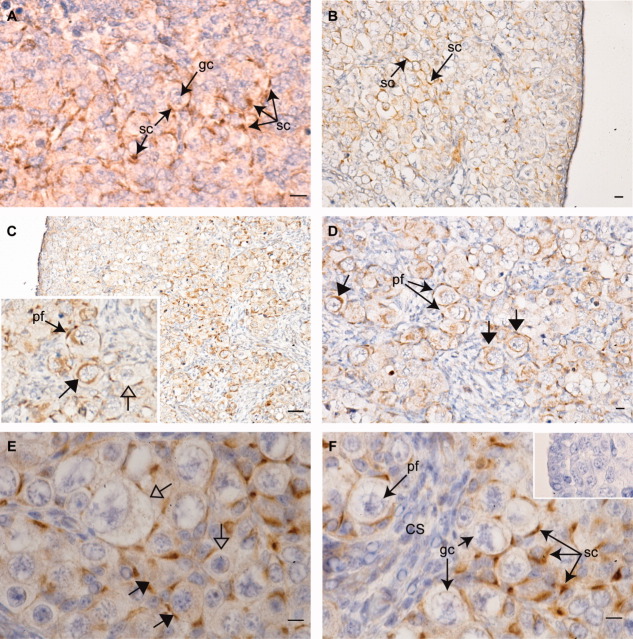
Immunohistochemical localization of brain-derived neurotrophic factor (BDNF) in the developing human fetal ovary. BDNF expression was detected at all stages examined. **A**: Expression of BDNF (brown staining) in somatic cells (sc) surrounding primordial germ cells (gc) in a first trimester (60 days gestation) human fetal ovary. **B,C**: At 14 (B) weeks and 20 weeks (C); BDNF is predominantly expressed in a corticomedullary gradient, with weak expression in somatic cells near the ovarian periphery, and intense expression in those interspersed between larger germ cells away from the periphery. C, inset: BDNF expression is detectable in the pregranulosa cells of primordial follicles (pf), and in some large germ cells (closed arrows), whereas others of comparable size and show no expression (open arrows). **D**: At 20 weeks; BDNF immunopositive germ cells are detectable within primordial follicles. **E**: At 18 weeks; BDNF expression varies between germ cells within the same nest, with immunopositive and immunonegative germ cells in existing close proximity. **F**: At 18 weeks; BDNF expression is strongest in somatic cells interspersed within germ cell nests and in primordial follicles. No expression is detectable in somatic cells within cell streams (cs). F, inset: negative control, primary antibody preincubated with immunizing peptide. Scale bars = 500 μm in A–D, 100 μm in E,F).

The distribution of NT4 protein in the human fetal ovary has been reported previously (Anderson et al.,^2002^); however, no comparable data exist on the expression of BDNF. We therefore performed immunohistochemistry to identify the site(s) of BDNF production in the developing human fetal ovary. BDNF was localized to the cytoplasm of somatic cells of the fetal ovary in all specimens/gestations examined, from 9 to 20 weeks gestational age (Fig. 2A–F). The distribution of BDNF within the ovary was found to be nonhomogeneous, however, with weaker expression detected in somatic cells associated with less mature germ cells at the periphery of the ovary, and more intense staining in somatic cells associated with larger, more mature germ cells toward the more central region of the organ in the second trimester (Fig. 2B,C). This was clearest at later gestations, after the onset of primordial follicle formation (Fig. 2C). Strong expression was detected in somatic cells interspersed within nests of germ cells, and in the pregranulosa cells of primordial follicles, but BDNF expression was very low/absent in somatic cells in streams between clusters (Fig. 2D,F). At later gestations, expression of BDNF could also be detected in the cytoplasm of some larger germ cells including those within newly formed primordial follicles (Fig. 2D,E) although variation in BDNF expression was apparent between adjacent germ cells within the same nest (Fig. 2E,F).

The pattern of BDNF protein expression in the somatic cells of the human fetal ovary is similar to that we have reported previously for NT4 (Anderson et al.,^2002^). The production of both NT4 and BDNF by somatic cells, and the expression of TrkB receptors by germ cells in the developing ovary, strongly suggests a role for somatic cell-derived TrkB ligands in regulating oocyte development. The expression of BDNF by germ cells at later gestations reveals additional levels of complexity in TrkB signaling in the human fetal ovary, indicating that germ cell fate may be regulated by both paracrine and autocrine neurotrophin signaling. Furthermore, the onset of BDNF expression in germ cells at this stage may explain part of the apparent increase in *BDNF* mRNA levels around the time of follicle formation detailed above.

In contrast to the situation in the human fetal ovary, we were unable to detect any expression of Bdnf protein in the developing mouse ovary by immunohistochemistry, although robust Bdnf expression was detectable in mouse brain as a positive control (data not shown). This result confirms the suggestion from our qRT-PCR data detailed above that Bdnf is expressed at an extremely low level, if at all, in the feto-neonatal mouse ovary.

### Activin A Selectively Regulates Expression of *BDNF* in the Human and *Ntf5* in the Mouse Ovary

**Fig. 3:**
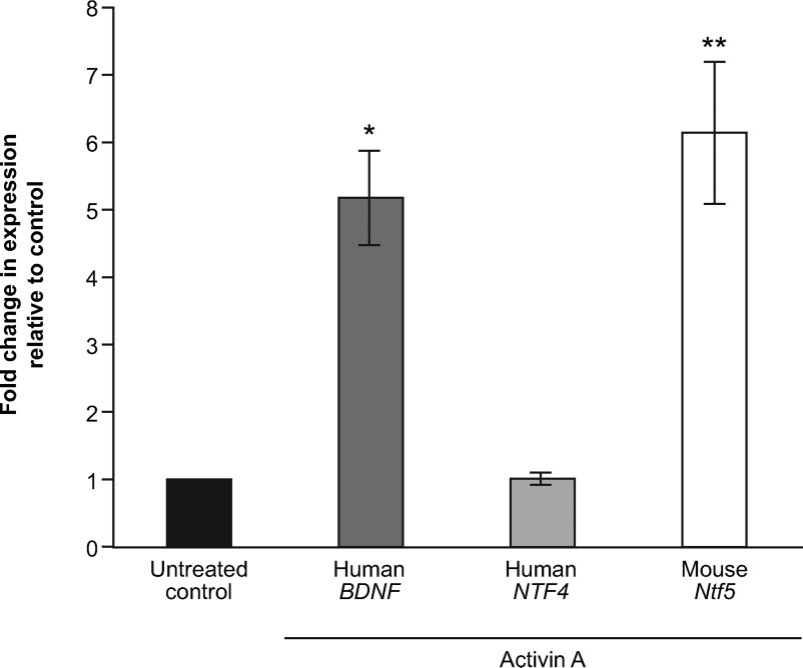
Selective regulation of *t*ropomyosin-*r*elated *k*inase (Trk) B ligand expression by Activin A in cultures of human fetal ovary and mouse neonatal ovarian somatic cells. Treatment of disaggregated second trimester human fetal ovaries (14–17 weeks gestational age, n = 5) with recombinant human activin A increased brain-derived neurotrophic factor (*BDNF*) expression five-fold relative to untreated controls (5.2 ± 0.7; *P* = 0.02), but had no effect on the expression of *NTF4* (1.01 ± 0.09, not significant). Comparable treatments of newborn mouse granulosa cells with activin for 5 days resulted in a six-fold (6.1 ± 1.05; *P* = 0.01; n = 4) increase in *Ntf5* gene expression. *Bdnf* transcript levels were too low to be reliably determined both in controls and after activin treatment.

The Transforming Growth Factor-β (TGF-β) superfamily member activin A is transiently expressed by oocytes in syncitial clusters, and is a central regulator of germ cell survival and primordial follicle formation in the feto-neonatal ovaries of both humans and rodents (Martins da Silva et al.,^2004^; Bristol-Gould et al.,^2006^). Activin influences germ cell behavior indirectly, by regulating the expression of reciprocal signals produced by neighboring somatic cells (Coutts et al.,^2008^; Childs and Anderson,^2009^), however, the identity of the factors produced by somatic cells that influence oocyte proliferation and survival in response to activin remain largely unknown. Given the localization of BDNF (this study) and NT4 (Anderson et al.,^2002^) to somatic cells interspersed between oocytes in germ cell nests, we postulated that these factors may represent good candidate factors for activin-regulated somatic cell-to-oocyte signaling molecules. To test this hypothesis, we performed cultures of disaggregated second trimester human fetal ovaries (14–17 weeks gestational age) in the presence or absence of recombinant human activin A for 18–24 hr and determined the expression of *BDNF* and *NTF4* by quantitative RT-PCR. These experiments demonstrated that activin A increased *BDNF* expression five-fold relative to untreated controls (5.2 ± 0.7; *P* = 0.02; n = 5), but had no effect on the expression of *NTF4* (1.01 ± 0.09, not significant, Fig. 3).

To determine whether the regulation of neurotrophin expression by activin is conserved between mouse and human, we cultured somatic cells from neonatal mouse ovary in the presence or absence of recombinant activin A, and assessed the effects on the expression of *Bdnf* and *Ntf5*. Treatment of somatic cells with recombinant activin for five days resulted in a six-fold (6.1 ± 1.05; *P* = 0.01; n = 4) increase in *Ntf5* gene expression (Fig. 3), whereas *Bdnf* transcript levels remained below the threshold required for reliable detection.

Although the specific TrkB ligand-encoding gene that is regulated by activin A appears to have diverged between humans and mice, it is notable that activin regulates the more highly expressed (*Ntf5* in mouse) or more regulated (*BDNF* in human) of the two TrkB neurotrophin genes suggesting that the relative physiological importance of the two ligands within the ovaries of humans and rodents may have been reversed. The finding that these two genes (*BDNF* in the human and *Ntf5* in the mouse) encoding signaling molecules with established roles in the regulation of germ cell survival and proliferation are downstream transcriptional targets of oocyte-derived activin signaling, supports our hypothesis that activin A acts to promote the expression of reciprocal signals that influence germ cell survival, and repress those which encourage oocyte maturation and follicle formation. Indeed, we have recently identified kit ligand as a another potential target of activin action in the human fetal ovary (Coutts et al.,^2008^; Childs and Anderson,^2009^), and it is likely that other such factors remain to be identified.

### Conclusions

Bi-directional communication between the germ and somatic cell compartments of the ovary is essential in the regulation of germ cell proliferation and survival, in orchestrating the breakdown of germ cell nests, and in the subsequent association of oocytes with pregranulosa cells that leads to the formation of primordial follicles. In support of recent data highlighting the importance of TrkB signaling in establishing the primordial follicle pool in mice (Spears et al.,^2003^; Kerr et al.,^2009^), the data presented here reveal differential expression of the TrkB ligands NT4 and BDNF, and species-specific differences between humans and mice in the regulation of TrkB ligand gene expression by activin A.

What then is the role of TrkB ligand signaling onto germ cells in the fetal ovary? The downstream targets of BDNF and NT4 action in the developing ovary are yet to be conclusively identified, although an increasing body of evidence suggests a role for these factors in preventing germ cell death. Pharmacological inhibition of all Trk receptor signaling with the inhibitor K252a impairs germ cell survival in both the human fetal ovary (Spears et al.,^2003^) and testis (Robinson et al.,^2003^). Mice carrying targeted disruptions of the *Ntrk2* (TrkB) locus display defects in germ cell survival and follicle formation and development (Spears et al.,^2003^; Kerr et al.,^2009^). Furthermore, the finding that both NT4 and BDNF can prevent apoptosis in human embryonic stem cells (Pyle et al.,^2006^), a cell type suggested to share many phenotypic similarities with early germ cells (Zwaka and Thomson,^2005^), adds weight to the hypothesis that TrkB ligands may be involved in regulating germ cell death. Indeed, given the onset of BDNF expression in the largest germ cells around the time of nest breakdown, and the variation in BDNF expression between germ cells within the same nest (Fig. 2C,F), it is tempting to speculate that BDNF/TrkB signaling may be involved in determining which germ cells are subject to the selective apoptosis that triggers germ cell nest breakdown leading to primordial follicle formation (Pepling and Spradling,^2001^). Work from our laboratory and others has demonstrated that activin A exerts both pro-proliferation and pro-survival effects on germ cells in the developing mammalian ovary (Martins da Silva et al.,^2004^; Bristol-Gould et al.,^2006^), and that these effects are mediated indirectly by modulating the expression of growth factors produced by neighboring somatic cells (Coutts et al.,^2008^; Childs and Anderson,^2009^). The finding that the expression of the genes encoding BDNF (in the human) and NT4 (in the mouse) are regulated by activin, suggests these factors may be downstream effectors of the positive effects of activin on germ cell development. A recent report has suggested that expression of components of the notch signaling pathway may be dysregulated in the ovaries of TrkB-null mice (Kerr et al.,^2009^). Given that notch-jagged signaling has recently been demonstrated to regulate the timing and extent of primordial follicle formation in the mouse (Trombly et al.,^2009^), this observation warrants further investigation. Coupled with our recent finding that the expression of *INHBA* (encoding the βA subunit of activin A) and *BDNF*, but not that of *NTF4*, is regulated by prostaglandin E2 (PGE_2_) in the human fetal ovary (Bayne et al.,^2009^), it appears that extensive cross-talk may exist between the signaling pathways regulating germ and somatic cell interactions around the time that follicle formation is initiated.

In summary, we have identified significant differences in the expression of the genes encoding the TrkB ligands NT4 and BDNF both within and between the developing ovaries of humans and mice, and these variations suggest that the relative levels of TrkB ligands may be significant in determining their effects. Furthermore, we have demonstrated selective transcriptional regulation by activin of the genes encoding BDNF in the human, and NT4 in the mouse, positioning these factors as potential somatic cell-derived components of a regulatory pathway that controls germ cell survival or maturation in response to oocyte-derived activin, and further expanding the repertoire of growth factors regulated by this protein. The results presented here illustrate the complexity and interdependency of growth factor signaling networks that mediate communication between the germ and somatic cell compartments around the time of primordial follicle formation. They also highlight the existence of major differences between humans and rodents in seemingly conserved signaling pathways during ovarian development, thus underlining the importance of studying human ovarian physiology directly rather than extrapolating solely from rodent models. Further elucidation of the factors regulating the onset and extent of primordial follicle formation, and their downstream targets, may provide insight into the pathogenesis of premature ovarian failure, and inform strategies to promote oocyte differentiation from germline stem cells in vitro for the study and treatment of this condition.

## EXPERIMENTAL PROCEDURES

### Animals

Mice (C57Bl6J) were housed in an environmentally controlled room on a 14-hr light, 10-hr dark photoperiod in accordance with UK legal requirements. To obtain staged embryos, females were housed with a proven male overnight and inspected the following morning for vaginal plugs. Any plugged females were determined to be at E0.5, with the day of birth then designated as P0.

### Collection of Human Fetal Tissues

Fetal ovaries were obtained from morphologically normal human fetuses (9–20 weeks gestational age) following elective medical termination of pregnancy. Informed consent was obtained and the study approved by the Lothian University Hospitals ethics committee. Pregnancies were terminated by treatment with mifepristone (200 mg orally) followed 48 hr later by misoprostol (800 μg, 3 hourly per vaginum). Gestational age was estimated by ultrasound scan before termination, and confirmed by direct measurement of foot length. Dissected ovaries were fixed in Bouins solution and processed into paraffin using standard methods (for immunohistochemistry, all gestations), frozen and stored at −80°C (for RNA extraction, 14–20 weeks gestation), or prepared for use in culture experiments as described below.

### RNA Isolation and cDNA Synthesis

Total RNA was extracted from frozen and cultured human fetal ovaries using the RNeasy Mini/Micro Kit (Qiagen, Crawley, UK) with on-column DNaseI digestion as per the manufacturer's instructions. Reverse transcription was performed using Expand Reverse Transcriptase (Roche Diagnostics, Lewes, UK) as described previously (Coutts et al.,^2008^). RNA from cultured mouse ovarian somatic cells was extracted using Qiashedders (Qiagen, Crawley, UK) with the RNeasy Micro Kit and subsequently reverse transcribed using a Quantitect Reverse Transcription Kit (Qiagen) according to the manufacturer's instructions.

### Quantitative RT-PCR

Analysis of TrkB ligand gene expression in human and mouse feto-neonatal ovaries was performed by qRT-PCR using a Lightcycler 1.0 instrument (Roche Diagnostics Ltd, Welwyn Garden City, UK) and SYBR Green PCR Master Mix (Qiagen), with melt curve analysis as described previously (Hartley et al.,^2002^; Coutts et al.,^2008^). Increasing dilutions (from 1:5 to 1:10,000) of fetal human or mouse ovary cDNA were used to generate standard curves of the number of cycles needed to yield a fluorescent signal above background against the log of relative concentration. The resulting slope of the curve is a measure of the efficiency of the PCR reaction (Hartley et al.,^2002^). The slopes of the standard curves are indicative of the efficiency of the PCR reaction and were all close to −3.3 (equivalent to 100% PCR efficiency or two-fold amplification per cycle), allowing quantification using the 2^−ΔΔCt^ method (Bayne et al.,^2009^). What small differences did exist were nevertheless taken into account when calculating the relative concentrations of each gene product, and adjusted for the relative molecular weight of each amplicon to give molar masses. Primer sequences and assays used are detailed in Table 1. Calculation of human *BDNF* and *NTF4* mRNA concentrations were made relative to *GAPDH* to allow comparisons between cDNAs. *Bdnf* and *Ntf5* levels were calculated relative to murine *Gapdh*. Analysis of murine *Ntf5* expression in response to activin treatment was performed using SYBR Green technology on an Opticon DNA Engine (MJ Research, MA, USA).

**Table 1:** qRT-PCR Primers and Primer Assays Used in This Study^a^

Protein	Species	Gene symbol	Forward primer (5′-3′)	Reverse primer (5′-3′)
BDNF	Human	*BDNF*	AACAATAAGGACGCAGACTT	TGCAGTCTTTTTGTCTGCCG
	Mouse	*Bdnf*	AACCATAAGGACGCGGACTT	TGCAGTCTTTTTATCTGCCG
NT4	Human	*NTF4*	Quantitect Hs_NTF4_1_SG primer assay^b^	
	Mouse	*Ntf5*	Quantitect Mm_Ntf5_1_SG primer assay^b^	
GAPDH	Human	*GAPDH*	GACATCAAGAAGGTGGTGAAGC	GTCCACCACCCTGTTGCTGTAG
	Mouse	*Gapdh*	GGGTGTGAACCACGAGAAAT	CCTTCCACAATGCCAAAGTT

a qRT-PCR, quantitative reverse transcriptase-polymerase chain reaction; BDNF, brain-derived neurotrophic factor; NT4, neurotrophin-4; GAPDH, glyceraldehyde-3-phosphate dehydrogenase.

b Obtained from QIAGEN (Crawley, UK).

### Immunohistochemistry

Immunohistochemistry was performed essentially as described previously (Martins da Silva et al.,^2004^) with the following modifications. After dewaxing, antigens were retrieved by pressure cooking in 0.01 M sodium citrate buffer for 5 min. Sections were blocked for 30 min in normal swine serum diluted 1:5 in Tris buffered saline (TBS; pH7.4) supplemented with 5% bovine serum albumin (BSA), followed by further blocks using the streptavidin/biotin blocking kit (Vector Labs, Peterborough, UK) with TBS washes between each step. Sections were incubated overnight at 4°C in anti-BDNF (Santa Cruz Biotech, Santa Cruz, CA) primary antibody (diluted 1:50 in blocking solution). The primary antibody was detected using biotinylated-swine anti-rabbit secondary antibody (DAKO, Cambridge, UK) diluted 1:500 in blocking solution for 30 min at room temperature, followed by a further incubation of 30 min with streptavidin-HRP (Vector Labs) diluted 1:1,000 in TBS. Bound antibodies were visualized using 3,3′-diaminobenzidine tetrahydrochloride (DAKO). Primary antibody preincubated in the presence of excess immunizing peptide (BDNF) was used as a negative control. Slides were counterstained with hemotoxylin, dehydrated and mounted, and photographed using a Provis microscope (Olympus, London, UK).

### Effect of Activin Treatment on Neurotrophin Expression

Human fetal ovaries (n = 5; 14–17 weeks gestational age) were disaggregated to a single cell suspension and cultured in the presence or absence of 100 ng/ml recombinant human activin A (R&D Systems, Abingdon, UK) for 18–24 hr exactly as described previously (Coutts et al.,^2008^). RNA extraction and cDNA synthesis were performed as described previously (Coutts et al.,^2008^), with determination of *BDNF* and *NTF4* expression by qRT-PCR performed as detailed above.

Ovaries were dissected from 8 P0 mice were placed into tubes containing 1 ml prewarmed Tryple Express (Invitrogen, Paisley, UK) and warmed at 37°C for 20 min. Ovaries were then pipetted vigorously until they had completely disaggregated. One milliliter control medium (Waymouth's medium (Invitrogen) supplemented with 0.3%BSA and 10 IU/10 μg/ml penicillin and streptomycin) was added and the suspension centrifuged at 2,000 *g* for 5 min. The cell pellet was resuspended in 100 μl of medium and distributed equally between 4 wells of a 24-well tissue culture plate, 2 wells containing 1 ml of control medium and 2 wells containing medium supplemented with 100 ng/ml activin A (R&D Systems). Cultures were maintained in a 5% CO_2_, 95% air incubator at 37°C. After 24 hr, a complete media change was performed to remove any remaining oocytes or cellular debris. The cultures were then incubated for a further 96 hr, with 500 μl medium exchanged after 48 hr. Cells were recovered by trypsinization using Tryple Express, followed by centrifugation for 15 min at 1,000 *g* and RNA extracted and analyzed for changes in *Ntf5* and *Bdnf* expression as detailed above.

### Statistics

RT-PCR data on TrkB ligand expression over gestational ranges in the human and mouse were analyzed by analysis of variance with post hoc Bonferroni tests. Paired *t*-tests were used to determine significance in changes in TrkB ligand expression in response to activin treatment.
